# Guide to sound teeth: a syllabus-informed instructional session to provide targeted insights into the history of dentistry

**DOI:** 10.5195/jmla.2026.2287

**Published:** 2026-04-01

**Authors:** Jessica R. Hollister, Keith Mages, Thomas Murphy, Meelin Dian Chin Kit-Wells

**Affiliations:** 1 jrhollis@buffalo.edu, Dental Liaison Librarian, University at Buffalo Libraries, Buffalo, NY; 2 kcmages@buffalo.edu, Curator, Robert L. Brown History of Medicine Collection, University at Buffalo Libraries, Buffalo, NY; 3 tpm23@buffalo.edu, Archivist, Robert L. Brown History of Medicine Collection, University at Buffalo Libraries, Buffalo, NY; 4 mc30@buffalo.edu, Clinical Associate Professor Emeritus, Department of Pediatric and Community Dentistry, University at Buffalo School of Dental Medicine, Buffalo, NY

**Keywords:** Dental history, Medical humanities, Interdisciplinary collaboration, Dental students

## Abstract

**Background:**

This case report details an exploratory instructional session for dental students led by librarian-instructors at the University at Buffalo. Using historical source materials from the Robert L. Brown History of Medicine Collection, an hour-long session was developed to introduce year-one dental students to the history of their profession and its ongoing collaboration with important clinical populations.

**Case Presentation:**

At the request of course faculty, the University at Buffalo's Dental Liaison Librarian, History of Medicine Curator, and History of Medicine Archivist were invited to develop and lead a session on the history of dentistry for a first-year course, Profession, Practice, and Community Dentistry (PDO 801). A core feature of this course is the introduction of students to eight underserved dental patient populations—referred to as “communities of focus.” To supplement student learning, library staff utilized the holdings of the Robert L. Brown History of Medicine Collection to bring together stories, artifacts, and printed materials that spoke not only to the history of the profession, but also to the history of the communities of focus. Thought prompts were developed to guide students during a textual analysis activity that analyzed representative materials.

**Conclusions:**

Overall, this interdisciplinary collaboration provided the opportunity to develop and implement a syllabus-informed historical instructional session that offered targeted insights into dentistry's past. Through guided discussions, hands-on exploration, and textual analysis of historic materials, instructors worked to inspire and educate participating dental students as they progress further along their path as providers of patient-forward care.

## BACKGROUND

Subject librarians to health sciences schools at academic institutions are uniquely positioned to form relationships with health sciences faculty and incorporate medical humanities pedagogies into health sciences curriculums [1, 2]. As repositories of historic primary source materials, special collections are also well suited to enable impactful, perhaps unexpected, discussions among health sciences students [3]. Together, academic libraries and special collections can support interdisciplinary approaches to medical humanities by providing physical and intellectual neutral spaces to facilitate more meaningful conversations beyond the influences of traditional departmental contexts [4]. The University Libraries system at the University at Buffalo consists of a liaison structure wherein librarians provide subject-specific services to departments and schools at the university, including a Dental Liaison Librarian to the School of Dental Medicine. Beyond that, the University Libraries is home to multiple special collections, such as the Robert L. Brown History of Medicine Collection. Building off the existing literature, the University at Buffalo’s Dental Liaison Librarian [JH] and the Curator [KM] and Archivist [TM] from the Robert L. Brown History of Medicine (HoM) Collection collaborated with a Clinical Assistant Professor of Pediatric and Community Dentistry in the School of Dental Medicine [CKW] to design a syllabus-informed session introducing first-year dental students to selected facets of dentistry’s professional history.

Initial conversations began at an informational booth operated at the 2024 Buffalo Niagara Dental Meeting. Staffed by JH, KM and TM, the booth featured a sampling of historical dental artifacts from HoM, including hand-painted lantern slides from the New York State Department of Education, a “Compound Magneto-Electric Machine” (purportedly able to painlessly extract teeth), thumb-operated dental drill, and a dental pelican and tooth key (historic extraction tools). These artifacts facilitated an engaging discussion with CKW, a meeting attendee, and culminated with an invitation for JH, KM, and TM, to infuse historical conversations into CKW’s *Profession, Practice, and Community Dentistry* (PDO 801) course.

## CASE PRESENTATION

### Overview of Course and Session

A key feature of *Profession, Practice, and Community Dentistry* (PDO 801) is early student exposure to clinical sites and patient care. Through this course, students are introduced to eight dental patient communities of focus (see *Table 1*). As part of their learning, students visit area clinical sites related to each of these communities. It was decided that the HoM would be included among these visited clinical sites. After gaining familiarity with the course syllabus, and the communities of focus, JH, KM, and TM designed a session that would feature historical dental materials aligned with stated course objectives and communities of focus.

**Table 1:** Communities of Focus

Community Name	Community Description and Key Oral Health Topics
Pre- and post-natal community	Concerns both parents and babies before and after childbirth and into early childhood. Sometimes includes extended caregivers. E.g., pregnancy gingivitis; maternal oral health; infant teething; non-nutritive sucking behaviors; Early Childhood Caries (ECC); person-centered, compassionate care.
Adolescent community	The period of accelerated biological growth, changes, and social role transitions between childhood and adulthood. E.g., dental caries; poor oral hygiene; dental anxiety; desire for esthetics; oral piercings; substance use; sexual health considerations; eating disorders; person-centered, compassionate care.
Intellectual and Developmental Disabilities community	Intellectual and Developmental Disabilities (IDD) are conditions that begin early in life and affect learning, communication, daily living skills, or physical development. E.g., oral hygiene capacity; dental caries; xerostomia; sensory, behavioral, communication, and mobility considerations; dietary habits; caregiver education; person-centered, compassionate care.
HPV medically compromised community	Individuals whose immune systems or overall health make them less able to fight infections, including the human papillomavirus (HPV). E.g., risk of HPV; preventative care; vaccination education; caregiver education; sore monitoring; person-centered, compassionate care.
Elderly and aging community	Adults, typically 65 years or older, whose health, mobility, or daily living abilities may change as they age. E.g., xerostomia; dental caries; gum disease and inflammation; oral cancer; medication interactions; cognitive decline; accessibility needs; person-centered compassionate care.
Native American community	Diverse groups of Indigenous peoples of the United States. E.g., ECC; barriers to access; water fluoridation; diabetes; periodontal disease; tobacco use; oral cancer; person-centered, compassionate care.
Refugee community	Individuals and families who have been forced to flee their home country due to war, violence, persecution, or threats to human rights and who cannot safely return. E.g., dental caries; barriers to access; trauma and stress; communication, cultural, financial, and language considerations; oral health literacy; dental anxiety; person-centered, compassionate care.
Homeless/unhoused community	Individuals and families who lack stable, safe, and consistent housing. E.g., barriers to access; untreated oral diseases and infections; pain management; xerostomia; substance use; oral cancer; person-centered, compassionate care.

Ultimately, an hour-long session with the following objective was developed:

Students will be introduced to the history of dental practice, with a particular focus on community outreach. Using historic books, artifacts, and archival collections, participants will be exposed to a variety of materials from the HoM collection and will work with facilitators to better understand how historical practices continue to impact contemporary dentistry.

To accommodate the course structure and total number of students, four identical sessions were held, with individual students attending on specific dates within their assigned group. Using this model, each student was able to visit the HoM once during the 2025 Spring Semester. Every session, students separated into groups for two stand-alone learning components, run simultaneously. Half of participants began their experience in the HoM’s reading room for a tour and general overview of dental history [KM]. The second group of students began with a hands-on analysis of historical texts related to the course’s communities of focus [JH, TM]. After 30-minutes, students switched locations, ensuring that everyone experienced both the museum tour and the textual analysis.

### Resources Selected

Building upon discussions with course faculty and the session objective, the HoM staff [KM, TM] worked to identify topical historic materials held within the collection. As the session consisted of two stand-alone components, two distinct sets of historic materials were selected. The first series of items were artifacts, archival, and printed materials meant to broadly illustrate the history of dentistry. Among those included were antique tooth-extraction instruments, such as dental pelicans and tooth keys, classic images and texts in the history of dentistry, such as Jan H. Steen’s 17^th^ c. etchings *The Charlatan* and *The Dentist* [5, 6]. Also featured was a stereoscope accompanied by stereographs from Cryer, Cunninham, and Waterston’s *Dental Stereoscopic Dissections of the Head and Neck* which represented a novel, early-modern mode of dental education [7]. See *Table 2.1* for an overview of items selected to illustrate the general history of the profession [5–26].

**Table 2:** Overview of Historic Materials Selected for Session

**2.1: Materials Selected to Illustrate the History of Dentistry as a Profession**	
Goepp, R. M. (1922). *Dental state board questions and answers*. Philadelphia, PA: W. B. Saunders Company.	Dental State Board questions and answers collected over the years from various dental journals, which are representative of questions asked by the State Board of Examiners in the United States.
Cryer, M. H., Cunningham, D. J., Waterston, D. (c.1900). *Dental stereoscopic dissections of the head and neck*. Meadville, PA: Keystone View Co.	A volume of stereoscopic cards that each showcase a different pair of images and descriptions that are designed to be placed into a stereoscope.
Stereoscope (early 20th c.). For viewing above.	An optical device designed to create the illusion of depth by presenting 2 slightly different images, so the brain combines them into a single 3-D image.
James, B., Callender, C., Buckingham, J. T. (1814). *A treatise on the management of the teeth*. Boston, MA: Charles Callender.	The first full-length book on dentistry published in the United States, and the first with a dental illustration.
Hunter, J., & Johnson, J. (1778). *A practical treatise on the diseases of the teeth : intended as a supplement to the natural history of those parts*. London, J. Johnson.	Early dentistry text concerning human teeth.
Tooth Extraction Instruments: Forceps (18th c.), Pelican (late 18th c.), Tooth Key (19th c.).	Forceps: A grasping instrument used to pull teeth. Pelican: A tool that was an improvement over the forceps with two claws and a two-sided hammer that acted as a lever to pull teeth. Tooth Key: A further advancement, this tool, which resembles a door key, provides greater leverage for faster extraction.
Visual depictions of historic tooth extractions; mounted, reprinted images included: Jan H. Steen. (c.1650). *The Charlatan*. Rijksmuseum, Amsterdam, Netherlands. Jan H. Steen. (1651). *The Dentist*. Royal Picture Gallery, The Hague, Netherlands. Gerard Dou. (1672). *Der Zahnarzt*. Gemäldegalerie Alte Meister, Dresden, Germany. David Teniers. (c.1660). *Der Zahnarzt*. Royal College of Surgeons of Edinburgh, Edinburgh, UK.	Prints of paintings depicting dentists extracting teeth, used to show both the perception of dentists and the brutality of extraction.
Dental Lantern Slides from New York Department of Education (early 20th c.; hand-colored, highlighting children demonstrating proper brushing technique).	Transparent photographic images mounted on glass plates for image projection.
**2.2: Historic Sources Analyzed by Students**	
Klein, H., Palmer, C. E. (1938). *Dental caries in American Indian children*. Washington, D.C.: U.S. Government Printing Office.	A report on dental caries in different Native American populations.
Keyes, F. A. (1919). Institutional dentistry (insane) report No. 4. *The Boston medical and surgical journal*, *180*(4), 89-93.	A report from Medfield State Hospital on how dentistry was practiced at the institution.
Spooner, S. (1838). *Guide to sound teeth, or A popular treatise on the teeth : illustrating the whole judicious management of these organs from infancy to old age* (2nd ed.). Collins, Keese, & Co.	A text that covers dental care from infancy to advanced age populations. Used as an example to show what about dentistry is the same or similar to modern dentistry.
Hogeboom, F. E. (1927). *Practical pedodontia, or juvenile operative dentistry and public health dentistry; an introductory text for students and practitioners of dentistry* (2d ed.). The C.V. Mosby Company.	A text that covers adolescent, perinatal/prenatal populations, and public health dentistry.
Cotton, H. A. (1921). *The defective, delinquent, and insane; the relation of focal infections to their causation, treatment, and prevention*. Princeton, N.J.: Princeton University Press.	A text that covers the Intellectual and Developmental Disabilities community. Draws a relationship between diseases/conditions and dental health.
Newmayer, S. W. (1924). *Medical and sanitary inspection of schools; for the health officer, the physician, the nurse and the teacher* (2nd ed.). Lea & Febiger.	A text that covers the Adolescent community and how dental practice is discussed in schools.
Gies, W. J. (1926). *Dental education in the United States and Canada; a report to the Carnegie Foundation for the advancement of teaching*. The Carnegie Foundation for the Advancement of Teaching.	A text demonstrating deficiencies in the dental care of Black and African American populations and statistics on early twentieth century dental schools in the U.S. and Canada.
Academy of Dentistry for the, H. (1963). Bulletin of Academy of Dentistry for the Handicapped. *Bulletin of Academy of Dentistry for the Handicapped.*	A text that covers the Intellectual and Developmental Disabilities community. Discusses the unique needs of patients with disabilities.
Brauer, J. C. (1939). *Dentistry for children*. P. Blakiston's Son & Co., Inc.	A text that covers dentistry in the Adolescent community and was used to demonstrate the techniques pediatric dentists use to engage children in the office.

The second series of selected items were historic source materials to be specifically analyzed by participants. Towards this end, representative items reflecting each of the course’s eight communities of focus were investigated. Unfortunately, the special collections team was unable to identify holdings specifically related to the provision of dental care to the unhoused or refugee populations.

Historic sources related to all other populations of focus were identified and made available for textual analysis. Among items selected for analysis were Dr. Henry A. Cotton’s (1921) *The defective, delinquent, and insane; the relation of focal infections to their causation, treatment, and prevention*, Klein & Palmer’s (1938) report for the U.S. Office of Indian Affairs, *Dental Caries in American Indian Children*, and the inaugural issue of the *Bulletin of Academy of Dentistry for the Handicapped* (1963-4), published by the eponymous professional organization [18, 22, 25]. See *Table 2.2* for an overview of materials consulted by students.

### Structure of Sessions and Pedagogy

Upon arrival, students were separated into two sections. Groups that began with the HoM component were first provided with a general overview of the collection. This included discussions on the founding of the University at Buffalo (1846) and the School of Dental Medicine (1892). Historic illustrations and photographs depicting the exterior and interior spaces of the university during these early eras were shown. Following this, a tour of the closed stacks was provided.

Next, students were brought into an adjacent instructional space to learn more about the history of dentistry as a profession, centered around the objects presented in *Table 2.1*. The spectacle and methods of pre-modern tooth extraction were illustrated with study of 17^th^ century etchings and contemporary extraction instruments such as dental forceps and pelicans. The evolution of dental education and outreach was touched upon with discussions regarding early licensing examinations, the dental dissection stereographs, and hand-colored glass plate slides from the New York State Department of Education depicting school children demonstrating proper brushing techniques. Several of these items were the same as those highlighted at the Buffalo Niagara Dental Meeting, described above, as they were well received at that event. Afterwards, students explored the collection on their own, as the instructor [KM] alerted the concurrent session instructors [JH, TM] that they were ready to switch groups.

During the textual analysis component, students were led to the HoM conference room space, which included an exhibit of an early 20th century dental clinic. Here, students were invited to choose a seat at the conference table aside 1 of 9 pre-selected historical source materials (see *Table 2.2*) pertaining to the communities of focus outlined in the syllabus (see *Table 1*). Inspired by Mages and Lohr’s medical humanities seminar, students were then oriented to the historic textual analysis activity structure: 12 to 15 minutes to analyze the source in front of them either individually or in small groups (left to student choice), followed by a 12 to 15-minute, moderated, full group discussion wherein students introduced their source and offered any insights or observations [3].

During this component, students were reminded that they would not be able to thoroughly review an entire source in the given time, however they were invited to consult a “thought prompt” handout (see *Table 3.1*) to guide their analyses. Designed to facilitate critical thinking and reveal throughlines between dental topics of the past and today, each thought prompt handout included a set of general questions (see *Table 3.1*) as well as a 1-2 source-specific questions (see *Table 3.2*). Handouts were placed with their respective source during set up. Thought prompts were developed using teaching with primary source pedagogies, such as those framed by the University of Maryland Baltimore County Special Collections at the Albin O. Kuhn Library and Gallery, asking students to meet the document, observe its parts, make sense of it, and use it as historical evidence [27].

**Table 3:** Thought Prompts Provided during Student Review of Materials

**3.1: General Guiding Thought Prompts**	**3.2: Source-Specific Thought Prompts Provided during Student Review of Materials**
Does anything surprise you?	According to the source, what is relationship between geography/environmental factors and dental caries? (*Dental Caries in American Indian Children*, 1938) [18]
Do you think this was forward-thinking at the time?	How is the dentist considering the unique needs of the patient? How are they not? (*Bulletin of Academy of Dentistry for the Handicapped*, 1963) [25]
Would you consider this patient-forward?	Are there relationships being drawn between diseases or conditions of the tooth/mouth and those with IDDs? (*The* d*efective, delinquent, and insane*, 1921) [22]
Who is the author, editor, or organization responsible for creating the source?	What is the relationship being drawn between pulling teeth and mental health? (*The* d*efective, delinquent, and insane*, 1921) [22]
Why was the resource created or published?	What is the significance of dental clinics being located in schools? (*Medical and sanitary inspection of schools*, 1924) [23]
What is the population of focus?	How are the techniques of pediatric dentists of the past similar or dissimilar to those of today? (*Dentistry for children*, 1939) [26]
What assumptions are being made about the population-in-question?	Does the sentiment of 'public health dentistry' in the text resonate today? In what ways? (*Practical pedodontia*, 1927) [21]
How does the setting, (i.e., time, place), inform the source?	How has practice changed? How has it remained the same? (*Guide to sound teeth*, 1838) [20]
Are there throughlines to dental education today?	What is the significance of the authors identifying Black and African American populations as a population in particular need? (*Dental education in the United States and Canada*, 1926) [24]
	Looking at the author’s 12 remedial suggestions for the Medfield State Hospital, how has dental practice changed? How has it remained the same? (*The Boston medical and surgical journal*, 1919) [19]

To further guide analysis, pre-set bookmarks were placed within each source to draw students’ attention to topics of potential interest. After the individual analysis, instructors [JH, TM] moderated full group discussions by going around the table and inviting each student or small group to introduce their source and anything within that caught their interest.

## DISCUSSION

### Student Reception to HoM Tour Component

Reflecting upon the HoM tour component of the sessions, it was noted that participants tended to be quieter at the beginning than at the end of this 30-minute portion. It is unknown if this relative lack of interaction with the instructor [KM] was due to the somewhat early hour (9:00AM), lack of interest in the more general portion of the tour, their presence in an unfamiliar site, or some combination of these factors. By the end of this portion, conversations and questions typically increased. This may be due to the introduction of artifacts more directly connected to dental history. When thinking specifically about these objects, discussions and demonstration of the tooth extraction instruments generated much interest. Students readily interacted with these pieces and often recoiled upon learning how they were used. Similarly, interactions with the stereoscope and the dissection stereographs were colorful, with exclamations often expressed when 3D images ‘snapped’ into focus for the viewing student. Such expressions align closely with earlier experiences of the instructors when introducing these specific materials to dental professionals.

One participant brought their own historic dental artifact to their session, in hopes of learning more about the piece, an early 20^th^ century circular brass cusp die-plate. Manufactured by L.D. Caulk Dental Depot, die-plates such as this were used in the molding of dental crowns. While the HoM holds other cusp die plates, this particular example was not among the collection’s holdings. Researching contemporary printed sources housed within the HoM, special collections staff were able to locate the same die-plate within a medical supply catalog from Lee S. Smith & Son (1905), as well as the methods and materials necessary to produce such crowns, reported within Henry A. Collett’s (1922) *Gold Shell Crowns and How to Make Them* [28, 29].

### Student Reception to Textual Analysis Component

Due to the interactive activity, reflections upon the textual analysis component of the session revealed more about student impact than the HoM tour component. On this front, the instructors [JH, KM, TM] have worked to discern which aspects were most successful, and which require refinement.

Similar to Rajagopalan et al., instructors [JH, TM] found that students were cautiously intrigued by the novelty of the sessions and may have considered working with historical primary sources a “break” from traditional didactic coursework [30]. The instructors worked to dispel any student uncertainty by clearly explaining the activity, how it relates to their course content, and acknowledging that the session may be different than their other dental school classes. To meet students at their respective learning and comfort levels, handouts of the thought prompts were provided to guide textual analysis, or as a “jumping off point” for those eager to unpack the content with their own observations. These served to foster engagement and dialogue amongst students, in preference of producing “correct” responses.

During the individual analysis portion of the session, many students preferred to view the guiding thought prompts as a structured assignment. They worked diligently to answer the proposed questions, rather than use them as a springboard for their own discovery. Thus, once these students answered the thought prompts on their handouts, they were content to sit and wait for the group discussion without further exploring their source. Whether this was due to the pressure of limited time, wanting to “complete” the assignment, lack of comfort with unstructured analysis, or some combination of the three, is unknown. Additionally, instructors noted a few disinterested participants chose to disengage once they saw there was no set assignment. Those few put little effort into their analyses or relied upon their fellow students to provide analyses on their behalf.

Differing learning styles were also reflected in the full group discussions, revealing unique insights and discoveries from the sources. When speaking to the group, most students preferred to introduce their source by answering the thought prompt questions exactly as they were indicated and not expanding beyond that. Other students answered portions of the thought prompts and instead used the group discussion to emphasize aspects of source material that piqued their interests. To further engage, instructors [JH, TM] responded to the students’ assessments with additional follow-up questions, where appropriate: “Did anything in this source surprise you?;” “What sparkled to you?;” “How is this similar or dissimilar to dentistry today?”. Such questions fostered open dialogue and encouraged students to draw their own comparisons between dentistry of the past and today. In one instance, a student excitedly shared a figure of a throat with diphtheria in Newmayer's (1924) *Medical and sanitary inspection of schools*, proclaiming that this was “the same figure” they were studying in preparation for an upcoming exam in a different class [23]. This enthusiasm reflects the sentiment expressed by students of Mages and Lohr in their medical humanities seminar, with one of their students stating the session was “Very interesting and relevant to our studies” [3].

Further, students would use the group discussions to highlight terms or practices from the historic sources they recognized, or phrases that resonated with them. For instance, from *Practical pedodontia*, a couple students emphasized the quote, “No one man has a monopoly on the best methods to care for children,” before describing how this outlook remains relevant in modern practice [21]. For both the *Medical and Sanitary Inspection of Schools* (1924) and *The Boston Medical and Surgical Journal* (1919), students contrasted the past presence of in-house dentists for preventative care in schools versus curative care in prisons and mental health facilities and posited how these past practices may continue to impact practice with these populations today [19, 23]. These student-led discoveries informed pedagogy, allowing the instructors [JH, TM] to expand their own knowledge and understanding of the sources, and to better put the sources in conversation with the dental student curriculum in subsequent sessions. Such discoveries also created avenues for conversation and feedback between instructors and students, allowing each group to learn from one another.

For future sessions, the instructors [JH, KM, TM] intend to work with the dental faculty to employ a flipped classroom approach using their university’s learning management system (LMS). As exemplified by Mages and Lohr’s 2017 study, students will be asked to browse digital excerpts of pre-selected, topical texts from the HoM collection via the LMS on their own time and ahead of the in-person session [3]. Upon their subsequent visit to the HoM collection, students will be able interact with the full, original source. The instructors anticipate that early exposure and interaction with historic materials may increase student interest and “buy-in” during the in-person session. Beyond that, students will have the opportunity to reconcile the digital snippet of the text with the full context of physical source, revealing new insights into the history of dentistry.

Although instructors did provide paper handouts of the thought prompts during the textual analysis activity, students were not required to complete these. The instructors also did not survey students after the session. Similar to Mages and Lohr, who viewed their medical humanities seminar as an outreach opportunity as much as a learning one, instructor priority was building positive and lasting rapport with the dental school and its constituents, while increasing the visibility of the HoM collection [3]. During future sessions, and with approval from the university’s institutional review board (IRB), the instructors plan to collect written student responses and post-class evaluations to better gauge impact and continue to improve class experience. Furthermore, as the instructors were unable to provide access to historical materials relating to refugee and unhoused communities during these sessions, a gap in HoM holdings has been identified. With this knowledge, HoM staff can target these areas for collection development initiatives.

Overall, this interdisciplinary collaboration provided the opportunity for the dental liaison librarian, HoM staff, and dental school faculty to develop and implement a syllabus-informed historical instructional session that provided targeted insights into dentistry’s past. Through the activities documented above, the instructors worked to inspire and educate dental students to better provide quality and historically informed patient care.

## Data Availability

There are no data associated with this article.
